# High-intensity interval training for heart failure with preserved ejection fraction: A protocol for systematic review and meta-analysis

**DOI:** 10.1097/MD.0000000000021062

**Published:** 2020-07-02

**Authors:** Sisi Zhang, Jingxian Zhang, Congying Liang, Xiaochuan Li, Xiaoping Meng

**Affiliations:** Cardiovascular and Cardiac Rehabilitation Department, The First Hospital of Changchun Chinese Medicine University, Changchun City, Jilin Province, China.

**Keywords:** high-intensity interval training, moderate-intensity continuous training heart failure with preserved ejection fraction, exercise intolerance, exercise capacity, cardiac rehabilitation

## Abstract

**Background:**

The benefits of high-intensity interval training (HIIT) are well-known, there is insufficient evidence about the effects of HIIT on heart failure with preserved ejection fraction (HFpEF).

**Method:**

Multiple databases include MEDLINE, PubMed, EMBASE, CINAHL, Web of Science, PEDro, Cochrane Library, and Google Scholar are used to search for randomized controlled trials investigating the effects of HIIT on HFpEF. All related articles published with the English language with no time limitation will be included. Two reviews independently conducted the selection, data extraction, and quality assessment. The primary outcome is exercise capacity. The secondary outcomes include quality of life (QoL), blood pressure (BP), ventricular function, and left ventricular diastolic function, symptom improvement, endothelial function, and arterial stiffness. Data analysis is performed with Review Manager Software (Version 5.3).

**Result:**

This systematic review and meta-analysis aim to evaluate the efficacy of HIIT on HFpEF, its outcome will provide reliable evidence for future studies.

**Conclusion:**

The findings of this study will be published in a related peer-reviewed journal.

**Registration number:**

INPLASY202050097

## 1 Introduction

As the 2016 European Society of Cardiology (ESC) guideline, left ventricular ejection fraction (LVEF) ≧50% is classified into HFpEF.^[1]^ For patients newly diagnosed with heart failure(HF), nearly half of them are heart failure with preserved ejection fraction and the prevalence is increasing over time.^[2,3]^ Some risk factors contribute to this prevalence, such as older age, female sex, obesity, hypertension, smoking, diabetes mellitus, coronary artery disease (CAD), et al. Most common symptoms include fatigue, weakness, dyspnea, orthopnea, paroxysmal nocturnal dyspnea, while exercise intolerance is the dominant one.^[4]^ Unlike heart failure with reduced ejection fraction (HFrEF), there is no effective therapeutic interventions have been proven beneficial. Diuretics, such as spironolactone are recommended by ESC to relieve fluid overload, while other medications did not get the recommendation. Exercise training as a novel therapeutic approach has been shown to improve aerobic capacity and quality of life in HFpEF.^[5,6]^ It is recommend combining endurance and resistance training for patients with HFpEF to improve exercise capacity, physical functioning, and diastolic function in ACC/AHA and ESC guidelines.^[1,7]^ Although exercise training has been recommended, the intensity does not get the consensus. Several decades ago, high-intensity interval training first introduced in CAD patients and chronic heart failure patients(HF).^[8,9]^ An intense curiosity of HIIT emerged in the American Heart Association in 2007 and now it is considered as an option exercise within a cardiac rehabilitation program.^[10,11]^ A recent meta-analysis also reported the superiority of HIIT compared with moderate-intensity continuous training (MICT) in patients with heart disease.^[12,13]^ However, there is insufficient evidence about the effects of HIIT on HFpEF and most published studies of HIIT in HF mainly focused on HFrEF. Thus HIIT demonstrates as an untested modality for exercise training in the HFpEF population. The purpose of this review is to evaluate the efficiency of HIIT in patients with HFpEF compared with MICT.

## 2 Method

This protocol is followed by preferred reporting items for systematic reviews and meta-analysis for protocol (PRISMA-P) guidelines.^[14]^ We registered this protocol on the

International Platform of Registered Systematic Review and Meta-analysis Protocols (registration no.INPLASY202050097).

### 2.1 Search strategy and literature sources

We will search for the literature from the following database: MEDLINE, PubMed, EMBASE, CINAHL, Web of Science, PEDro, Cochrane Library, and Google Scholar. The specific search strategy is shown in Table 1.

**Table 1:**
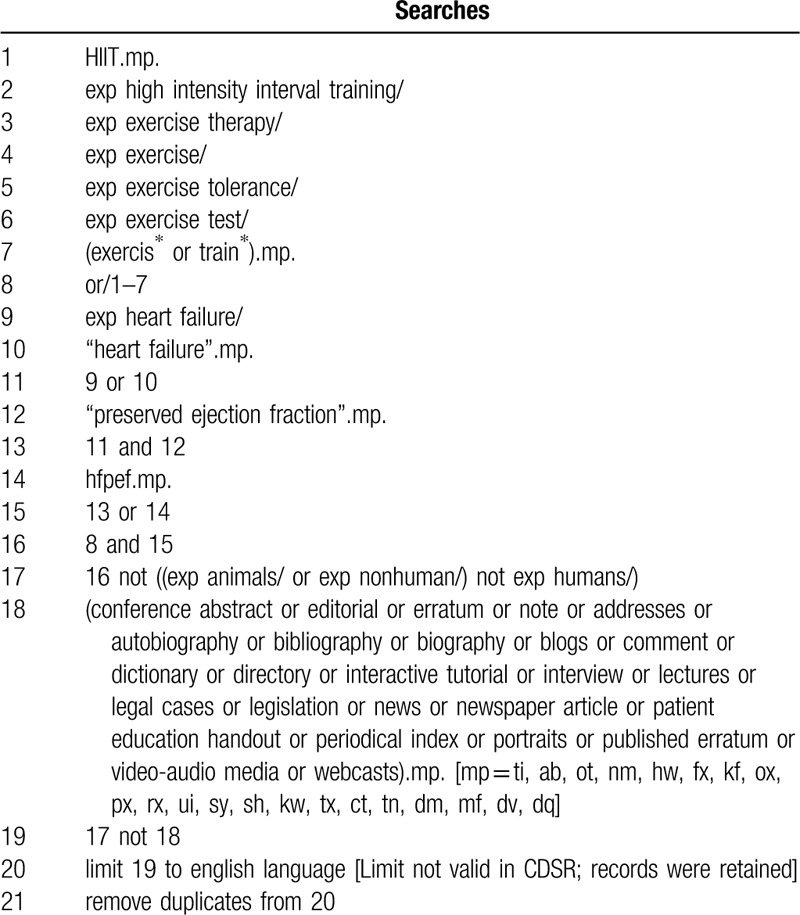
The search strategy of this systematic review.

### 2.2 Criteria

#### 2.2.1 Types of included studies

We only include randomized clinical trials or randomized control trials (RCT) published with the English language. None randomized control trials, books, theses, conference presentations, dissertations, abstracts, letters, editorial papers, non-human, systematic review and meta-analysis, study protocol, unpublished and non-English researches are excluded.

#### 2.2.2 Types of participants

Adults (age ≥18 years) with HFpEF (left ventricular ejection fraction > 50%) or diastolic heart failure (E/e’ > 15 or other diagnostic criteria as consensus by ESC) will be included, without the restriction of gender, nationality, and ethnicity.^[15]^

#### 2.2.3 Types of intervention

Interventions groups refer to high-intensity interval training (HIIT), the high-intensity is defined as achieving an 80% to 90% peak heart rate (PHR).^[16]^

#### 2.2.4 Comparator

The control group is MICT, which is defined as achieving between 64% and 76% PHR according to the American College of Sports Medicine's guidelines.^[17]^

#### 2.2.5 Types of outcomes

The primary outcome is exercise capacity, which is assess by cardiopulmonary exercise test (CPET) or 6-minute walking test (6MWT). The secondary outcomes are the following: quality of life (QoL), blood pressure (BP), ventricular function and left ventricular diastolic function, symptom improvement, endothelial function, and arterial stiffness

### 2.3 Study selection

**Figure 1:**
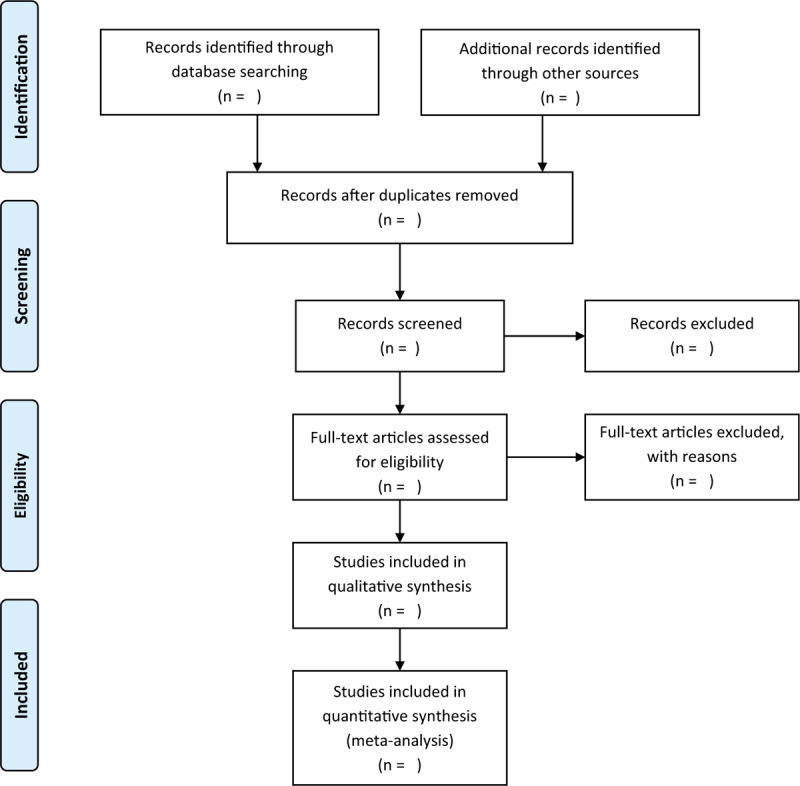
The flow diagram for the search and session progress.

Assessing the studies identified through a systematic search will be done in 2 steps in succession. First, 2 investigators screen literature that meets the inclusion criteria based on the title and the abstracts independently and blindly. Second, the selected studies will be further screened based on full texts. The entire screening process will be done independently and in duplicate. The disagreement between the two investigators in each stage is resolved by discussion and consensus; if the agreement is not achievable, a senior author is consulted until a consensus is reached. The flow diagram for the search and selection process is developed using the preferred reporting items for systematic reviews and meta-analyses (PRISMA) guidelines (shown in Fig. 1).^[18]^

### 2.4 Data extraction

Eligible full-text will be included for data extraction and further analysis by 2 reviewers. We will use a standard data extraction forms: general characteristics of the study (name of the first author, year of publication and country), sample size, describe of HIIT, characteristics of the intervention and control group, number and characteristics of participants in each group, rates of missing data, duration, length/intensity, outcomes. The data extraction process was performed independently and discrepancies discussed with a third reviewer until consensus was reached.

### 2.5 Quality assessment of included studies

The quality of studies included in the systematic review is scored using the PEDro scale from Physiotherapy Evidence Database by 2 dependent reviewers. PEDro is a useful tool related to assessing the quality of physical therapy and rehabilitation trials.^[19]^

### 2.6 Data synthesis and Statistical analysis

After data extraction, 2 reviewers will decide which outcome will be included in the meta-analysis, which is performed with Review Manager Software (Version 5.3).^[20]^ If studies contain incomplete data

Or missing data, a descriptive analysis will be made. The pooled-effect as the difference between HIIT and MICT from baseline to the endpoint was calculated and presented with the weighted mean difference (WMD) and a 95% confidence interval (CI). *P* value < .05 is considered statistically significant.

Heterogeneity between studies is assessed by the χ^2^ test and I^2^ test. I^2^ > 50% is considered high heterogeneity, then we adapt a random-effects model for data analysis.^[21]^

### 2.7 Sensitivity analysis

Sensitivity analysis will be performed to evaluate the reliability of results based on sample size, the methodological quality of the included studies. We will repeat the meta-analysis if it is necessary.

### 2.8 Grading the quality of evidence

The quality of the evidence will be performed by the grading of recommendations assessment, development, and evaluation (GRADE) system, which is classified into four levels, high, moderate, low and very low quality.^[22]^

## 3 Discussion

To our knowledge, this is the first systematic review and meta-analysis protocol about HIIT on HFpEF. The results will evaluate whether HIIT is superior to MIAC for patients with HFpEF. It will provide more evidence for future studies as to which intensity is the best option for HFpEF. However, there are also some limitations exist, firstly, we find that the definition of HFpEF lack of a uniform standard. ESC defines EF≧50% as HFpEF, while ACC/AHA states the diagnosis criterion as EF ≧40%. Heterogeneity will come from the different intensity of HIIT and evaluation standard in the different studies. Secondly, we only include studies published with the English language, other languages will be excluded.

## Author contribution

**Conceptualization:** Sisi Zhang, Xiaoping Meng

**Data curation:** Jingxian Zhang.

**Formal analysis:** Jingxian Zhang.

**Methodology:** Congying Liang.

**Project administration:** Xiaochuan Li.

**Supervision:** Xiaoping Meng.

**Writing – original draft:** Sisi Zhang.
